# Response to “complexity of interstitial lung disease disproportionality signal analysis in pharmacovigilance databases”

**DOI:** 10.1016/j.eclinm.2025.103451

**Published:** 2025-08-20

**Authors:** Zijun Zhu, Yongxin Li, Jiuda Zhao

**Affiliations:** Breast Disease Diagnosis and Treatment Center, Affiliated Hospital of Qinghai University, Affiliated Cancer Hospital of Qinghai University, Xining, China

We appreciate our colleagues' attention to our work and the opportunity to clarify several points.

Firstly, we selected the narrow standardized MedDRA query (SMQ) “interstitial lung disease (ILD)” in our study primarily because it focuses on preferred terms (PTs) directly associated with ILD, thereby reducing false positives and enhancing signal detection accuracy. In contrast, while the broad SMQ encompasses a wider range of PTs, it may include terms unrelated to novel antineoplastic agents, such as antisynthetase syndrome, goodpasture's syndrome, and complications of transplanted lung. These PTs are typically caused by other factors and are not directly linked to the use of novel antineoplastic drugs.

In our analysis, each PT was treated as an independent unit for signal detection. This choice is due to the fact that ILD is a complex adverse reaction that can manifest through multiple PTs. By analyzing PTs individually, we can more precisely capture ILD-related signals, especially when utilizing SMQ. This approach is both common and appropriate in pharmacovigilance research, as it offers higher precision for identifying specific adverse event types.^1^^,^^2^ We acknowledge our colleagues' valid concern that treating each PT as a separate event might inflate report numbers. To address this, we conducted an additional disproportionality analysis using only “interstitial lung disease” as the PT, which further strengthens the credibility of our findings and more accurately reflects the specific relationship between ILD and novel antineoplastic drugs. Regarding the inconsistency in the total number of events (a + b + c + d) across different drugs in eTable 3, we understand this may raise concerns and would like to clarify. In pharmacovigilance disproportionality analysis, it is standard practice to construct contingency tables independently for each drug. Consequently, the total number of events may vary due to differences in report numbers and event types for each drug in the database, reflecting the unique characteristics of each drug in real-world monitoring data.^3^ We agree that in future studies and reports, we should more clearly define the unit of analysis in the methods to prevent any confusion regarding data comparability.

We fully concur with our colleagues that spontaneous reporting databases do not allow for the scientifically rigorous calculation of traditional Hazard Ratios (HR) as seen in cohort studies. In eFigure 2, the Cox proportional hazards model was used solely as an exploratory tool to compare relative differences in the frequency of adverse reaction reports across different drug categories, not as a risk estimate based on follow-up time. To avoid misinterpretation, we deliberately refrained from using terms like “risk” in our results and instead employed phrases such as “higher/lower odds of reporting” to describe the observed outcomes.^4^ We recognize the limitations of this exploratory analysis and will exercise greater caution in selecting statistical methods in future research.

Finally, we analyzed both the FAERS and JADER databases separately and compared signal patterns across databases to minimize the impact of regional bias. While we attempted to adjust for potential confounding through multivariate regression modeling, we acknowledge that inherent limitations of spontaneous reporting systems may prevent complete elimination of biases like the Weber effect and treatment-line confounding. Regarding the stratification of ILD subtypes, we note that the SMQ for ILD includes a large number of PTs. Conducting time-to-onset analysis for each PT and drug class separately would significantly fragment the dataset and obscure overall trends. Therefore, our study focused on the broader differences in time-to-onset of ILD among different novel antineoplastic drug classes, aiming to reflect real-world clinical differences more effectively.

We deeply appreciate the best practice recommendations provided in the letter and believe these methods are invaluable for enhancing the robustness and clinical relevance of drug-ILD signal analyses.

## Contributors

ZZ, YL, and JZ contributed to critically reviewing or revising the manuscript and gave final approval for submission.

## Declaration of interests

All authors have declared no conflicts of interest.
